# Low-Temperature Thermally Reduced Molybdenum Disulfide as a Pt-Free Counter Electrode for Dye-Sensitized Solar Cells

**DOI:** 10.1186/s11671-015-1156-0

**Published:** 2015-11-17

**Authors:** Che-Hsien Lin, Chuen-Horng Tsai, Fan-Gang Tseng, Yang-Yen Yu, Hsuan-Chung Wu, Chien-Kuo Hsieh

**Affiliations:** Department of Engineering and System Science, National Tsing Hua University, Hsinchu, 30013 Taiwan, Republic of China; Department of Materials Engineering, Ming Chi University of Technology, 84 Gungjuan Rd., Taishan District, New Taipei City, 24301 Taiwan, Republic of China

**Keywords:** Molybdenum disulfide, Thermal reduction, Counter electrode, Dye-sensitized solar cells

## Abstract

A two-dimensional nanostructure of molybdenum disulfide (MoS_2_) thin film exposed layered nanosheet was prepared by a low-temperature thermally reduced (TR) method on a fluorine-doped tin oxide (FTO) glass substrate as a platinum (Pt)-free and highly electrocatalytic counter electrode (CE) for dye-sensitized solar cells (DSSCs). Thermogravimetric analysis (TGA) results show that the MoS_2_ sulfidization temperature was approximately 300 °C. X-ray photoelectron spectroscopy (XPS), high-resolution transmission electron microscopy (HRTEM), and X-ray diffraction (XRD) indicate that the stoichiometry and crystallization of MoS_2_ were more complete at higher temperatures; however, these temperatures reduce the number of edge-plane active sites in the short-range-order nanostructure. Accordingly, the DSSCs with 300 °C annealed TR-MoS_2_ CE exhibited an excellent photovoltaic conversion efficiency (PCE) of 6.351 %, up to 91.7 % of which is obtained using the conventional TD-Pt CE (PCE = 6.929 %). The temperature of thermal reaction and the molar ratio of reaction precursors were found to significantly influence the resulting stoichiometry and crystallization of MoS_2_ nanosheets, thus affecting DSSCs’ performance.

## Background

Since the first demonstration of dye-sensitized solar cells (DSSCs) by O’Regan and Grätzel [1], much attention has been paid to these third-generation solar cells due to their low cost, easy fabrication, high photo conversion efficiency, and environmental friendliness [2–5]. A DSSC typically comprises of a wide-band semiconductor (usually TiO_2_) coated with dye molecules on a transparent conductive glass as a working electrode (WE), an electrolyte-containing iodide/triiodide (I^−^/I_3_^−^) redox couple, and a counter electrode (CE), usually deposited platinum (Pt) on the transparent conductive glass. Pt is conventionally used as the CE catalyst in DSSCs to regenerate the electrolyte redox couple and collect electrons to complete the circuit. However, because the high cost and scarcity of Pt greatly restrict the commercial production of DSSCs, the development of low-cost, good electrical conductivity, and high-electrocatalyst CE materials is highly desired to provide an economic solution for high-performance DSSCs.

Stimulated by the outstanding electrochemical activity of graphene, two-dimensional (2D) nanomaterials have attracted great attention in recent years [5–8]. Transition metal dichalcogenides (TMDCs), MX_2_, (M = Nb, Ta, Mo, W; X = S, Se, Te), have received much interest due to their 2D layered nanostructures, which are analogous to the graphene structure [9–11]. As a typical TMDC, the layer-dependent properties of molybdenum disulfide (MoS_2_) have recently attracted considerable attention due to their great potential in the electrochemical fields of catalysis [9, 12], lithium-ion batteries [13–15], hydrogen evolution [9, 16, 17], and DSSCs [18, 19]. MoS_2_ is composed of three stacked atomic layers (a Mo layer sandwiched between two S layers, S–Mo–S) and held together through van der Walls interactions [20].

However, MoS_2_ tends to form zero-dimensional fullerene-like nanoparticles or one-dimensional nanotubes during the synthetic process [21, 22]. Therefore, an efficient way to synthesize 2D layer-nanostructured MoS_2_ is to use graphene or carbon nanotubes (CNTs) as a template substrate [10, 16, 18, 23]. Although significant efforts have been devoted to the preparation of 2D layer-nanostructured MoS_2_, including scotch tape-based micromechanical exfoliation [24], liquid exfoliation [25–28], hydrothermal synthesis [14, 29], physical vapor deposition [30, 31], and chemical vapor deposition [32, 33], the easy synthesis of 2D layer-nanostructured MoS_2_ at low temperatures by template-free approaches under mild conditions still remains a challenge [34, 35]. Additionally, the electrochemical activities of MoS_2_ were correlated with the number of catalytically active edge sites [9, 12, 17, 36], for the reason that controlling the nanostructures with more edge sites at the atomic scale is an effective strategy to gain an effective MoS_2_ catalyst. In this study, we produce an easy, thermally reduced (TR) MoS_2_ nanosheet thin film on fluorine-doped tin oxide (FTO) glass at low temperature that provides the number of edge-plane active sites in the short-range-order nanostructure of MoS_2_ nanosheets, and demonstrates good catalytic performance compared with conventional Pt CE DSSCs.

## Methods

### Preparations of the Molybdenum Disulfide Counter Electrodes

A FTO transparent glass (TEC-7, 2.2 mm, Hartford) substrate was ultrasonically cleaned sequentially in detergent, acetone (overnight), distilled water (DI water, 1 h), and ethanol (1 h). Ammonium tetrathiomolybdate ((NH_4_)_2_MoS_4_) powder (ProChem Inc., 99.99 % purity; 0.8 g) was added to 20 mL of N,N-dimethylformide (DMF) to form a 4 wt% solution. The solution was then sonicated for 1 day before use [28]. Furthermore, the dispersed solution was coated on FTO glass by spin coating at 1600 rpm to control the thickness and flatness of the film. The substrate was then dried in air for 1 h and annealed in an H_2_/Ar = 1:9 gas mixture at various temperatures for 45 min to obtain thermally reduced molybdenum sulfide (TR-MoS_x_) samples. The annealing temperatures for the MoS_2_ phase transformation in this study are 250, 300, and 350 °C. The thermally deposited platinum (TD-Pt) CE was prepared as a reference electrode by thermal reduction, which was carried out by dropping a H_2_PtCl_6_ isopropanol solution on an FTO glass substrate annealed at 450 °C for 20 min [37].

### Fabrication of the TiO_2_ Working Electrode

The working electrode utilized the same FTO glass coated with nanocrystalline TiO_2_ using the print-screen method; the area and thickness of the TiO_2_ film were about 0.28 cm^2^ and 10 μm, respectively. The TiO_2_ WE was then gradually sintered to 550 °C in ambient air for 30 min before being slowly cooled at room temperature (RT). After calcination, the TiO_2_ WE was then immersed in a N719 (Solaronix) solution (3 × 10^−3^ M in a 1:1 volumetric mixture of acetonitrile and *tert*-Butylalcohol) at RT for 24 h. Following the dye adsorption process, the dye-adsorbed TiO_2_ WE was washed with acetonitrile to remove the remaining dye and dried at RT for a few seconds.

### Fabrication of DSSC Devices

The efficiency of the TR-MoS_x_ CEs as well as the standard TD-Pt CE DSSC devices were quantitatively compared. The dye-adsorbed TiO_2_ WE was future assembled with a CE into a sandwiched configuration and sealed with a 60-μm hot-melt surlyn (SX1170-60, Solaronix) by heating at 100 °C for a few seconds. The DSSC device was fabricated by drilling two holes on the CE and injecting an iodide-based electrolyte (AN-50, Solaronix) in the space between the electrodes after the assembling process. Finally, the holes on the CE were sealed after the electrolyte injection. DSSC devices were then illuminated by a class A quality solar simulator with a light intensity of 100 mW cm^−2^ (AM 1.5), which was calibrated with a standard silicon cell.

### Characterizations of Molybdenum Sulfide Counter Electrodes

In order to investigate the phase transformation and chemical states of the low-temperature thermally reduced MoS_2_, thermogravimetric analysis (TGA) was conducted using a thermogravimetric analyzer (TGA Q50 V20.10 Build 36, USA) with a heating rate of 5 °C min^−1^ in ambient Nitrogen. X-ray photoelectron spectroscopy (XPS) was conducted using a PHI Quantera SXM/AES 650 (ULVAC-PHI INC., Japan.) equipment with a hemispherical electron analyzer and a scanning monochromated Al Kα (hv = 1486.6 eV) X-ray source to study the chemical states of Mo and S of the prepared MoS_x_ annealing samples. The XPS curve-fitting program, XPSPEAK 4.1, was used for peak de-convolution and assignment of binding energies, which was referenced to the adventitious C1s peak at 284.6 eV. For spectrum analysis, the background signal was subtracted by Shirley’s method, and the curve fitting was performed by using a Gaussian–Lorentzian peak after Shirley background correction. Raman spectra were collected with a confocal micro-Raman spectroscopy (LABRAM HR 800 UV, Japan) using a 514-nm Ar^+^ laser source with a spot size of approximately 1 μm. The surface morphology of the prepared MoS_x_ annealing samples was examined by using the field emission scanning electron microscope (FESEM, JEOL, JSM-6330F, Japan). The nanostructures of MoS_2_ were examined by using the transmission electron microscope (TEM, JEOL-2100F, Japan) equipped with EDS to determine the elements contained in the samples. X-ray diffraction (XRD, PANalytical-X‘Pert PRO MPD) with a CuKα radiation of 0.1541 nm was used to determine the crystallinities of the films.

According to our previous studies [3, 4], cyclic voltammetry (CV) measurements and the electrochemical impedance spectroscopy (EIS) were carried out to examine electrochemical properties. CV measurements were used to measure electrochemical redox ability using a potentiostat/galvanostat (PGSTAT 302N, Autolab, Eco Chemie, Netherlands) in a three-electrode configuration. Platinum wire and an Ag/AgCl electrode were used as the counter and reference electrodes, respectively. The solution used for CV measurements contained 1 mM I_2_, 10 mM LiI, and 0.1 M LiClO_4_ in acetonitrile. EIS spectra were obtained using the aforementioned potentiostat/galvanostat equipped with a frequency response analysis (FRA) module; EIS results were analyzed using an equivalent circuit model with Autolab FRA software (v4.9, EcoChemie B.V.). The frequencies used in the scan ranged from 10^6^ to 10^−2^ Hz, and an applied voltage of 10 mV was used.

In addition, Tafel polarization measurements were carried out using the potentiostat/galvanostat equipped with a linear polarization module to examine the electrocatalytic activity at the electrolyte–electrode interface. Both EIS and Tafel polarization measurements were obtained using symmetrical devices in the dark.

The photocurrent–voltage characteristics of DSSC devices were measured under simulated solar illumination (AM 1.5, 100 mW cm^−2^, Oriel 91160, Newport Corporation, USA), equipped with an AM 1.5G filter (Oriel 81088A, Newport Corporation, USA) and a 300-W xenon lamp (Oriel 6258, Newport Corporation, USA). The simulated incident light intensity was calibrated using a reference Si cell (calibrated at NREL, PVM-81).

## Results and Discussion

### Composition and Morphologies

**Fig. 1:**
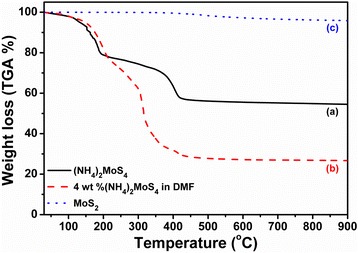
Thermogravimetric analysis (TGA) curves at 5 °C min^−1^ for the thermal decomposition in N_2_ atmosphere of (*a*) (NH_4_)_2_MoS_4_, (*b*) (NH_4_)_2_MoS_4_ dispersed in DMF, and (*c*) MoS_2_ powder

In this study, our homemade CVD system served as an important tool for the TR-MoS_x_ annealing at low temperatures. TGA curves for the three complexes, (NH_4_)_2_MoS_4_, 4 wt% (NH_4_)_2_MoS_4_ in DMF, and pure MoS_2_ powder, as shown in Fig. 1, were analyzed to identify the MoS_2_ sulfidization temperature region. Quantitative data derived from the TGA curves are summarized in Table 1. As we can see from curve (a) in Fig. 1, the (NH_4_)_2_MoS_4_ monomer decomposition was similar to a previous report [38]; there are two TGA regions in curve (a). The first is a sharp step from RT to 200 °C that corresponds to the loss of ammonia and hydrogen sulfide according to the following reaction:

**Table 1:** Thermal analysis data for the thiomolybdates decomposition in N_2_

Compound	TG region	Temperature range (°C)	Probable product	Experimental weight loss (%)
(NH_4_)_2_MoS_4_	1	RT −200	MoS_3_	21.08
	2	200–900	MoS_2_	45.50
(NH_4_)_2_MoS_4_ + DMF	1	RT −225	MoS_3_(+DMF)	24.51
	2	225–900	MoS_2_	73.31
MoS_2_	1	RT −900	MoS_2_	<1

1 $$ {\left({\mathrm{NH}}_4\right)}_2{\mathrm{MoS}}_4\to 2{\mathrm{NH}}_3 + {\mathrm{H}}_2\mathrm{S} + {\mathrm{MoS}}_3 $$

The second decomposition occurs from 200 to 420 °C, indicating the MoS_2_ phase transformation according to Eq.(2).

2 $$ {\mathrm{MoS}}_3 + \mathrm{H}\to {\mathrm{MoS}}_2 + {\mathrm{H}}_2\mathrm{S} $$

The (NH_4_)_2_MoS_4_ precursor dispersed in DMF was also analyzed by TGA (curve (b) in Fig. 1); the most weight loss occurs in the temperature range from 220 °C to 450 °C, which also indicates the MoS_2_ phase transformation. Commercial MoS_2_ powder was also used as a reference (curve (c) in Fig. 1) that shows a broad temperature region (RT to 900 °C) and great thermal stability [39]. For future study, we carried out the three annealing conditions in our homemade furnace thermal CVD in H_2_ mixed gas (H_2_/Ar = 1:9) at 350, 300, and 250 °C.

**Fig. 2:**
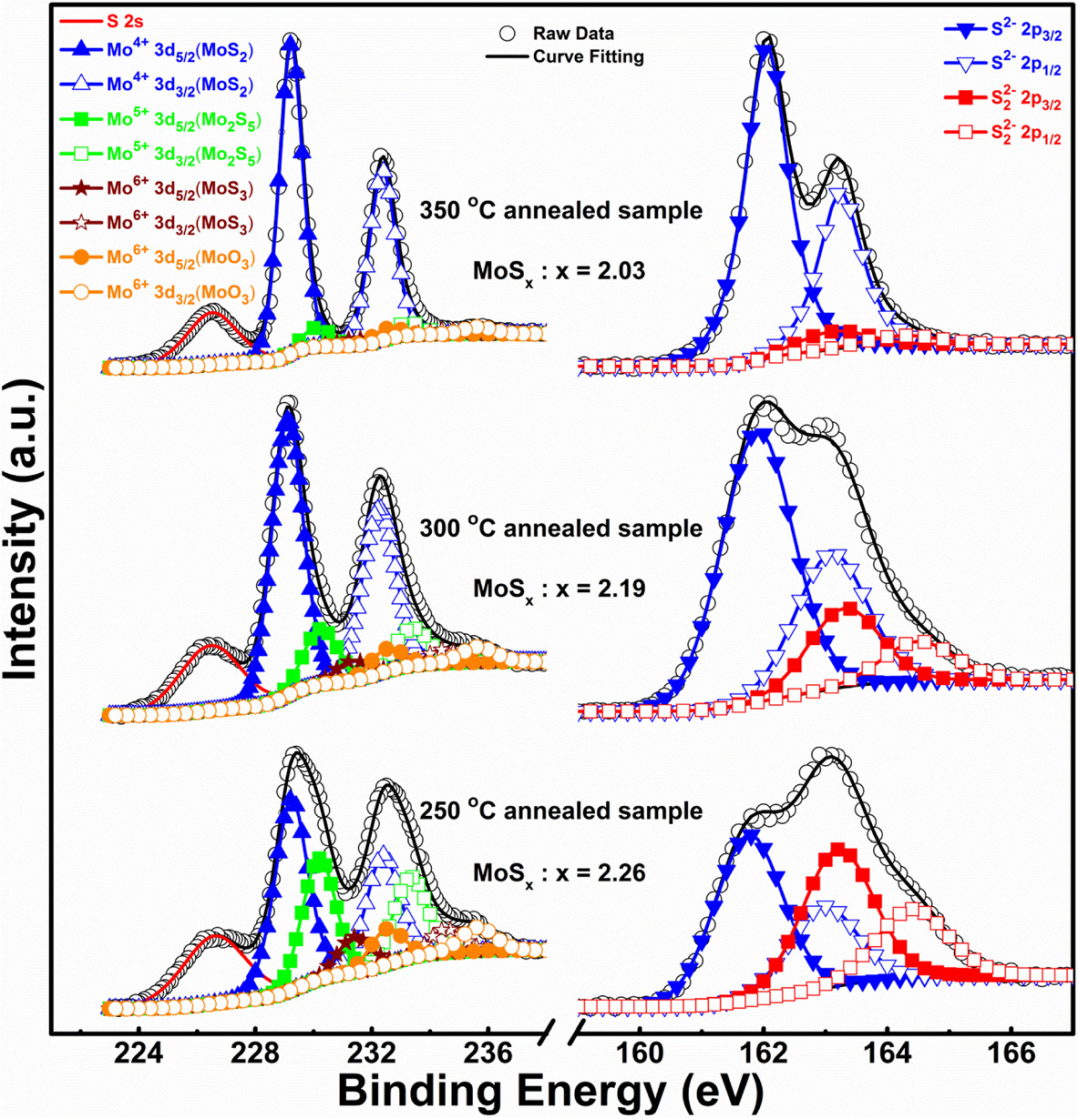
The Mo 3d and S 2p peak in the XPS spectra of the TR-MoS_x_ annealed at 350, 300, and 250 °C

In this study, we focused on the non-stoichiometric chemical compositions of TR-MoS_x_ thin films in different temperature regions; the relationship of temperature with the performance was studied by XPS at various temperatures. Figure 2 shows the XPS spectrum of the TR-MoS_x_ samples annealed at 350, 300, and 250 °C; the left side and right side of Fig. 2 show the chemical states of Mo 3d and S 2p orbitals, respectively. The peak positions, intensities (atomic percentage), and the stoichiometric ratio (S/Mo) are also given in Table 2.

**Table 2:** The Mo 3d and S 2p peak positions, atomic percentages, and *x* values of the TR-MoS_x_ samples annealed at 250, 300, and 350 °C

Peak and identity		Fitting of Mo 3d and S 2p peak binding energy (eV) (atomic percentage (%))		
		250 °C	300 °C	350 °C
Mo^4+^ 3d_5/2_	MoS_2_	229.23(31.2)	229.10(44.4)	229.22(54.0)
Mo^4+^ 3d_3/2_		232.37(18.1)	232.24(27.6)	232.36(32.6)
Mo^5+^ 3d_5/2_	Mo_2_S_5_	230.18(19.2)	230.23(9.7)	230.10(4.2)
Mo^5+^ 3d_3/2_		233.32(12.8)	233.37(6.4)	233.24(2.8)
Mo^6+^ 3d_5/2_	MoS_3_	231.40(5.6)	231.30(3.4)	231.40(1.7)
Mo^6+^ 3d_3/2_		234.54(3.8)	234.44(2.3)	234.54(1.1)
Mo^6+^ 3d_5/2_	MoO_3_	232.50(5.6)	232.50(3.7)	232.50(2.2)
Mo^6+^ 3d_3/2_		235.64(3.8)	235.64(2.5)	235.64(1.5)
S^2−^ 2p_3/2_	MoS_2_	161.76(35.7)	161.90(51.8)	162.05(61.7)
S^2−^ 2p_1/2_		162.95(17.9)	163.09(25.9)	163.24(30.9)
S_2_^2−^ 2p_3/2_	–	163.20(30.9)	163.30(14.9)	163.10(4.9)
S_2_^2−^ 2p_1/2_		164.39(15.5)	164.49(7.4)	164.29(2.5)
S/Mo ratio (*x* values)		2.26	2.19	2.03

The TR-MoS_x_ annealed at 350 °C exhibits two main peaks of Mo 3d spectra at 229.22 and 232.36 eV that correspond to Mo^4+^ 3d_5/2_ and Mo^4+^ 3d_3/2_ orbitals, revealing that the Mo^4+^ state is dominant in the 350 °C annealed sample and indicating the formation of MoS_2_ [12]. Additional peaks are observed at 162.05 and 163.24 eV, which correspond to the known S 2p^2−^_3/2_ and S 2p^2−^_1/2_ MoS_2_ doublet peaks, respectively [12]. The stoichiometric ratio (S/Mo) quantified by relative sensitivity factors (RSF) from the respective integrated peak area of XPS spectra is close to 2.03, also indicating that the structure of the 350 °C annealed sample is MoS_2_. Whereas the annealing temperature is lowered to 250 °C, in addition to that the XPS peaks of the MoS_2_ structure, other deconvoluted peaks need the concern. The peaks at 230.18 eV (Mo^5+^ 3d_5/2_) and 233.32 eV (Mo^5+^ 3d_3/2_), 231.40 eV (Mo^6+^ 3d_5/2_) and 234.54 eV (Mo^6+^ 3d_3/2_), 232.50 eV (Mo^6+^ 3d_5/2_) and 235.64 eV (Mo^6+^ 3d_3/2_), representing the Mo 3d_5/2_ and Mo 3d_3/2_ of the three valence states can be assigned to Mo_2_S_5_, MoS_3_, and MoO_3_, respectively [12, 40, 41]. Meanwhile, the binding energy at 163.20 eV (S_2_^2−^ 2p_3/2_) and 164.39 eV (S_2_^2−^ 2p_1/2_) might represent to the intermediate product of Mo_2_S_5_ and the MoS_3_ with a formula of [Mo^4+^ (S_2_)^2−^S^2−^] [42, 43]. It is worthwhile to mention that the MoS_2_ fraction decrement is nearly linear with annealing temperature down to 250 °C, and it becomes gradual at lower temperatures. These results suggest an incomplete MoS_2_ phase transformation at lower annealing temperature, which is consistent with our TGA results. The stoichiometric ratio (S/Mo) estimated from the 250 and 300 °C annealing sample were 2.26 and 2.19, respectively. In addition, compared with the 250 °C annealing sample, note that the line width of MoS_2_ peaks becomes progressively stronger and narrower for annealing temperatures above 300 °C.

**Fig. 3:**
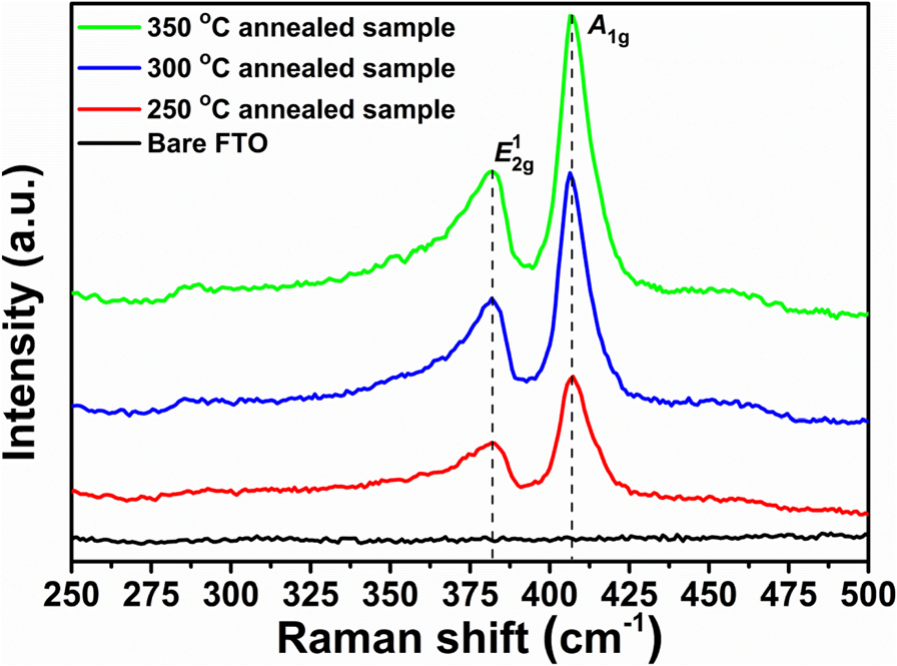
Raman spectra of TR-MoS_x_ samples annealed at various temperatures recorded using a 514-nm excitation wavelength

The Raman spectrums of the synthesized samples are shown in Fig. 3. All annealing samples showed the two prominent Raman peaks of MoS_2_ at about 381 cm^−1^ (*E*^1^_2g_) and 406 cm^−1^ (*A*_1g_) [44]. The *E*^1^_2g_ mode corresponds to the in-plane vibration from two S atoms with respect to the Mo atom in opposite vibration; the *A*_1g_ mode is associated with the out-of-plane vibration of only S atoms along the plane directions [45]. In addition, the observation of *E*^1^_2g_ and *A*_1g_ Raman peaks at 381 and 406 cm^−1^ suggested the presence of multi-layered MoS_2_ nanosheets [44, 45]. Meanwhile, the peak intensity and full width at half maximum (FWHM) of MoS_2_ becomes stronger and narrower as the annealing temperature up to 300 °C, that suggested the complete and genuine MoS_2_ structure.

**Fig. 4:**
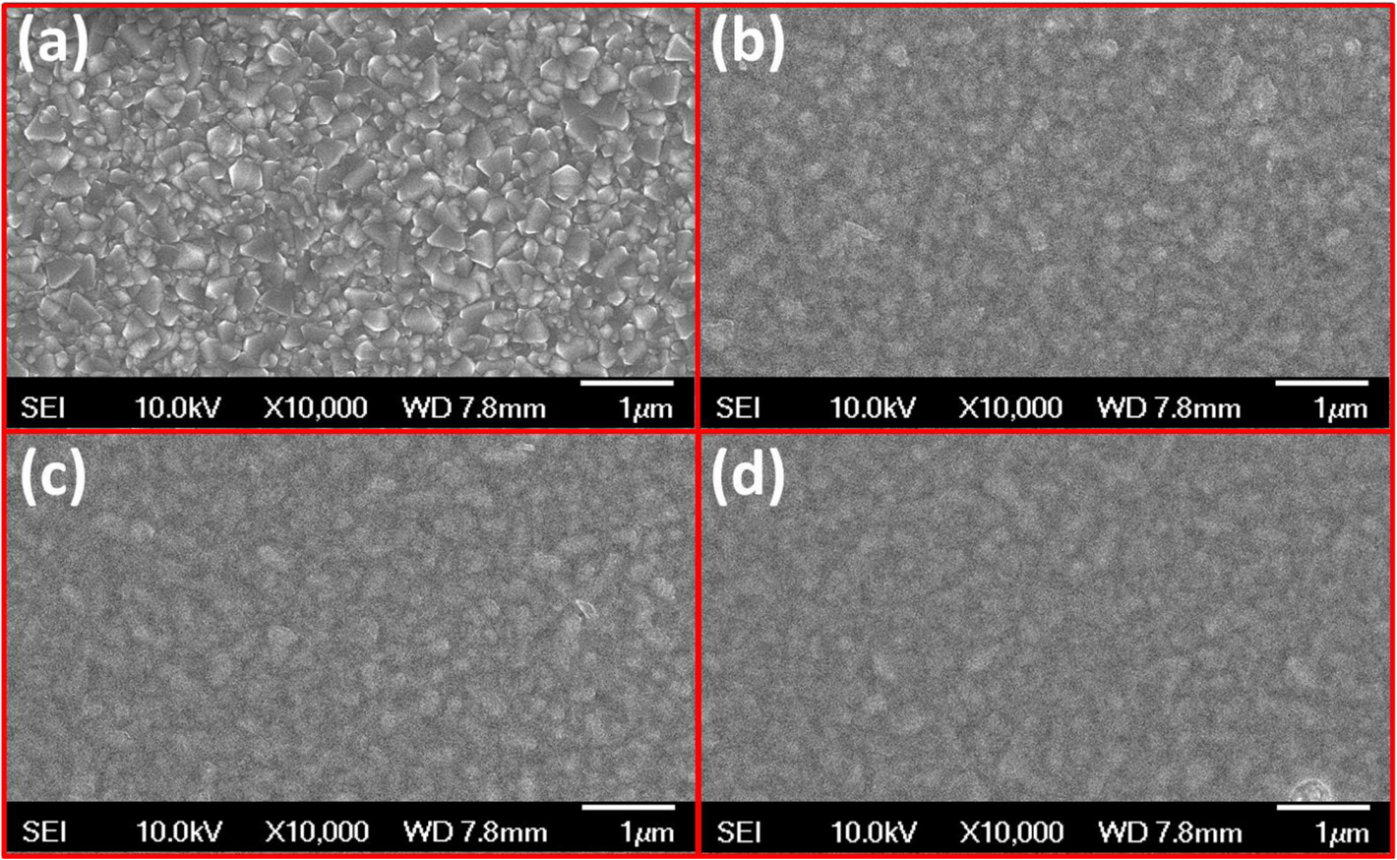
The SEM images of the **a** bare FTO and the TR-MoSx samples annealed at **b** 250, **c** 300, and **d** 350 °C, respectively

Figure 4 shows the SEM images that correspond to the bare FTO substrate and the TR-MoSx samples annealed at 250, 300, and 350, respectively. Obviously, all the prepared TR-MoSx electrodes showed the film-like morphology on the surface of FTO substrate, which was prepared by spin coating technology and controlled the flatness of the film.

**Fig. 5:**
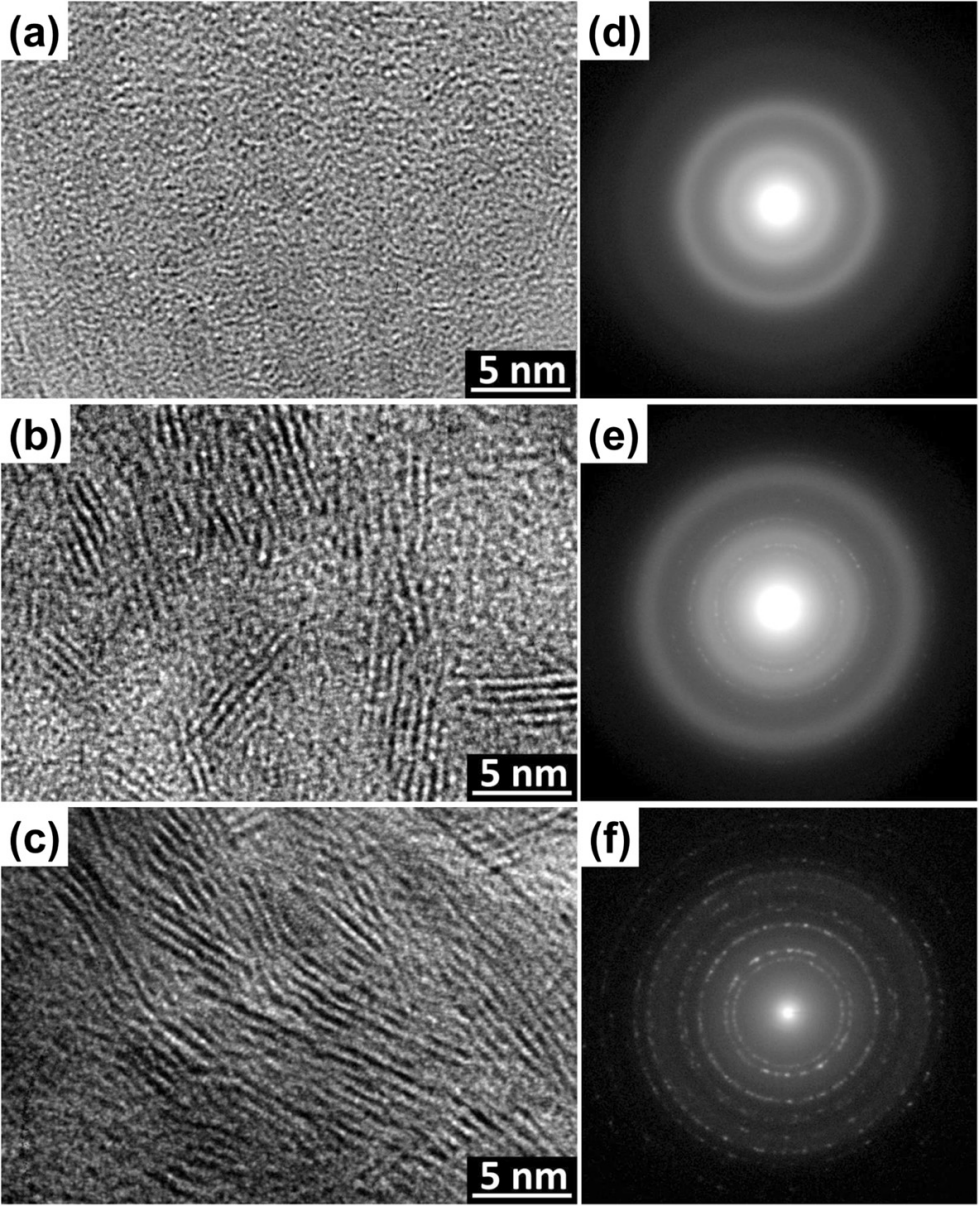
**a**–**c** TEM images of the TR-MoS_x_ annealed at 250, 300, and 350 °C and **d**–**f** the corresponding diffraction patterns, respectively

**Fig. 6:**
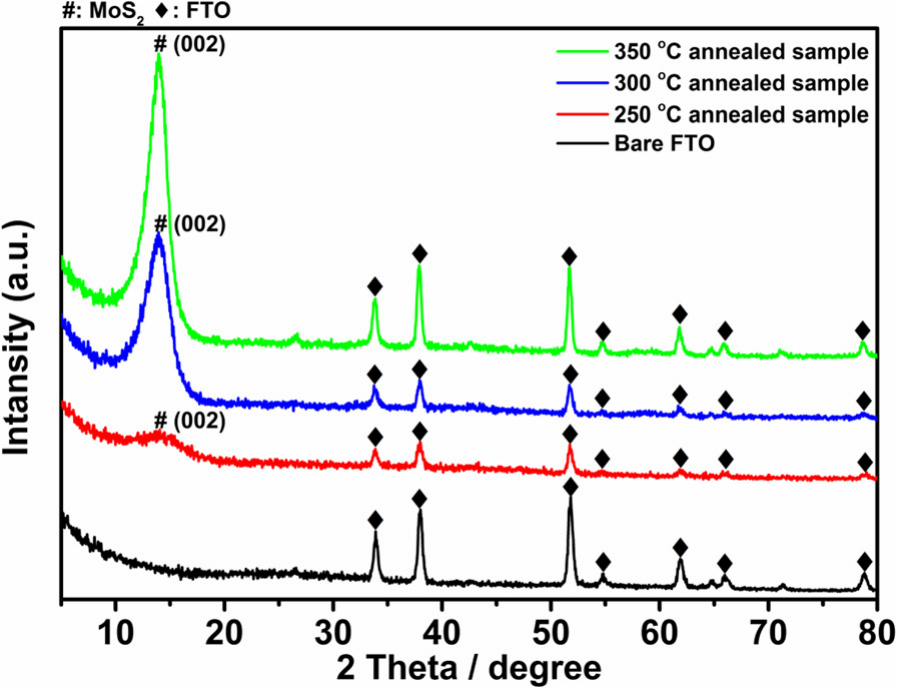
The XRD results of the FTO glass and the TR-MoSx samples annealed at 250, 300, and 350 °C, respectively

Figure 5 shows high-resolution transmission electron microscopy (HRTEM) images and the corresponding selected area electron diffraction (SAED) of the annealed samples at 250, 300, and 350 °C. Figure 5a, d shows the HRTEM image and SAED of the MoS_x_ annealed at 250 °C, respectively. Because the major phase is MoS_3_ at 250 °C, we can confirm that the MoS_3_ synthesized at this temperature is noncrystalline, which is consistent with a previous report [18]. Fig. 5b shows the separated layer-nanostructured MoS_2_, formed after the MoS_3_ was converted to crystallized MoS_2_ at 300 °C. The lateral size of the crystal domain in the independent MoS_2_ nanosheets is approximately few nanometers, corresponding to 3–5 layers as seen in Fig. 5b; Fig. 5e shows the SAED of Fig. 5b. These short-range-order structure of MoS_2_ nanosheets played a number of important roles: (i) the edge-planes of the MoS_2_ nanosheet structure provided a large number of active sites for redox reactions [12], (ii) the separation and independence of the MoS_2_ nanosheet allowed the I_3_^−^ ions to easily diffuse to the active sites of the edge-planes for the reduction reaction, and (iii) the independent MoS_2_ nanosheets increased the specific surface area available to promote the charge-transfer rate. Accordingly, the interlayer distance of the MoS_2_ nanosheets in this study was about 6 Å; this distance corresponds to the spacing between the (002) basal planes of MoS_2_, similar to a previous study [15, 45]. Fig. 5c, f show the HRTEM image and SAED of MoS_x_ annealed at 350 °C, respectively. The structure of the MoS_2_ nanosheets becomes more complete and reduces the number of the edge-planes of the MoS_2_ nanosheets, as shown in Fig. 5c. Due to this short-range-order structure, fewer edge-planes of active sites can be used in the redox reactions. By comparing Fig. 5f, e with the HRTEM images at different annealing temperatures and their corresponding SAED patterns, we can determine whether the MoS_2_ crystallization was more complete at higher temperature. To further confirm the identity and structure of the annealed sample, XRD measurements were carried out. Figure 6 shows the XRD pattern of the TR-MoSx samples annealed at 350, 300, and 250 °C, respectively. The diffraction peak (#) of (002) that comes from the basal planes of MoS_2_ can be observed. On the other hand, compared with the 250 °C annealed sample, the peak intensity and FWHM of MoS_2_ become more stronger and narrower as the annealing temperature reach to 300 °C; it suggested that the MoS_2_ crystallization was more complete at higher temperature. From the TEM and XRD results, it is noted that the layered MoS_2_ nanosheet thin films synthesized on FTO with sulfurization are actually polycrystalline when the annealing temperature exceeds 300 °C; the grain growth occurred at 350 °C to form the long-range-order MoS_2_ nanosheets.

### Electrocatalytic Properties

**Fig. 7:**
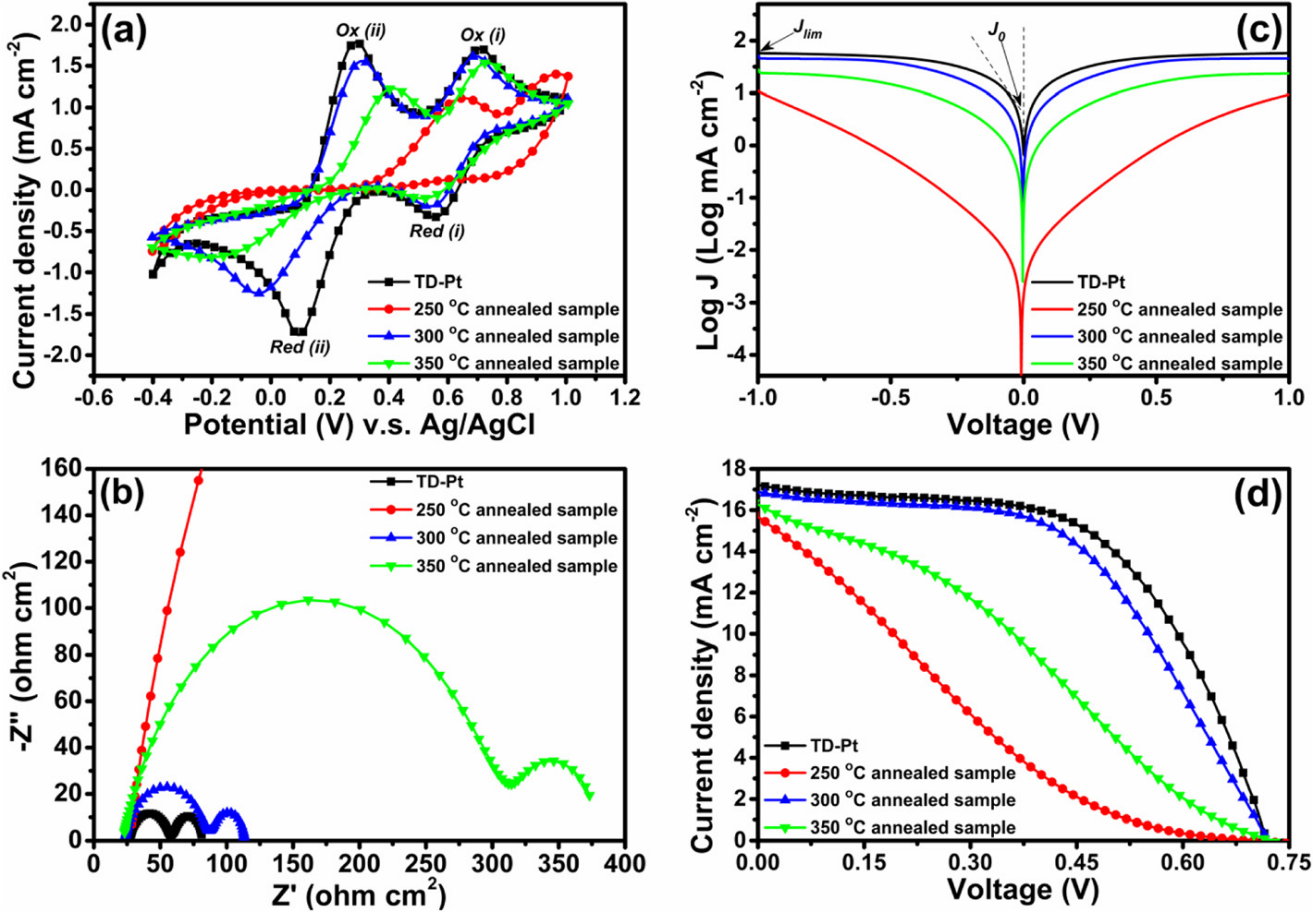
Electrocatalytic properties and characterization of the DSSCs assembled with various CEs. **a** CV curves. **b** Nyquist plots. **c** Tafel-polarization curves. and **d** Photocurrent–voltage curves

In order to estimate the electrocatalytic performance of the TR-MoS_x_ toward I_3_^−^ reduction, CV analysis was performed using a potential interval ranging from −0.4 to 1.0 vs Ag/AgCl and a scan rate of 50 mV s^−1^. Figure 7a shows the results of the CV measurements in the I^−^/I_3_^−^ system based on the TD-Pt CE and TR-MoS_x_ CEs annealed at 250 °C, 300 °C and 350 °C. Typically, there are two pairs of redox peaks in the cyclic voltammogram. The relatively positive pair (Ox (i) and Red (i)) corresponds to the redox of I_2_/I_3_^−^(Eq. (3)). The other pair (Ox (ii) and Red (ii)) is associated with the redox of I_3_^−^/I^−^, as presented in Eq. (4).

3 $$ 3{\mathrm{I}}_2 + 2{\mathrm{e}}^{-} = {{2\mathrm{I}}_3}^{-} $$

4 $$ {{\mathrm{I}}_3}^{-} + 2{\mathrm{e}}^{-}=3{\mathrm{I}}^{-} $$

The anodic peak current (*I*_pa_) and the cathodic peak current (*I*_pc_) corresponded to the oxidation of I^−^ ions and the reduction of I_3_^−^ ions, respectively. The magnitude of *I*_pc_ corresponded to the catalytic activation of a C.E. for I_3_^−^ reduction in a DSSC [3]. In addition, the peak to peak voltage separation between the anodic and the cathodic peaks (*E*_pp_) can be considered to the redox barrier of I_3_^−^/I^−^ redox couples. Therefore, the higher *I*_pc_ and lower *E*_pp_ values means the better electrocatalytic activity of CEs in DSSC [19]. From Fig. 7 (a), it can be observed that the TD-Pt and TR-MoS_x_ CEs exhibit two redox pairs, whereas no significant peak is observed when the annealing temperature is below 300 °C. This indicates that our TR-MoS_x_ annealed at 300 °C exhibits similar electrocatalytic activity to the TD-Pt CE, while the noncrystalline TR-MoS_x_ CE annealed at 250 °C provides poor electrocatalytic activity. Furthermore, the current density of the redox peaks for the TR-MoS_x_ CE annealed at 300 °C is higher than those annealed at 250 and 350 °C. In other words, compared to the other conditions, the 300 °C annealed sample can exhibits the highest *I*_pc_ and lowest *E*_pp_ that toward the best I_3_^−^ ion electrochemical reduction performance.

To further investigate the electrocatalytic properties, EIS was carried out using symmetrical cells comprising two identical TD-Pt CEs and various TR-MoS_x_ CEs annealed at 250, 300, and 350 °C, as shown in Fig. 7b. The corresponding EIS parameters obtained from a Nyquist plot are summarized in Table 3, in which *R*_s_ corresponds to the series resistance of the electrolyte and electrodes and *R*_ct_ is the charge-transfer resistance at the electrolyte–electrode interface. From Fig. 7b and Table 3, the *R*_ct_ values for the TD-Pt CE and the TR-MoS_x_ CEs annealed at 300 and 350 °C are 14.98, 30.98, and 141.41 Ω cm^2^, respectively; no *R*_ct_ value is identified for MoS_x_ annealed at 250 °C because the *R*_ct_ was too big to fit. We herein suggest that the noncrystalline MoS_3_ (annealed at 250 °C) exhibits a poor conductivity due to insufficient energy for sulfidization to produce MoS_2_, which is consistent with CV results (Fig. 7a).

**Table 3:** Photovoltaic parameters of the DSSCs based on various CEs and electrochemical parameters from EIS and Tafel measurements

Counter electrode	*R*_s_/Ω cm^2^	*R*_ct_/Ω cm^2^	*J*_0_/mA cm^−2^	*J*_sc_/mA cm^−2^	*V*_oc_/*V*	FF	*η*(%)
TD-Pt film	27.17	14.98	4.78	17.056 ± 0.075	0.724 ± 0.003	0.557 ± 0.007	6.929 ± 0.063
250 °C annealed sample	28.58	–	0.01	15.442 ± 0.118	0.709 ± 0.004	0.175 ± 0.002	1.917 ± 0.026
300 °C annealed sample	23.89	30.98	2.54	16.905 ± 0.013	0.727 ± 0.003	0.517 ± 0.005	6.351 ± 0.045
350 °C annealed sample	23.13	141.41	0.54	16.063 ± 0.251	0.725 ± 0.005	0.299 ± 0.005	3.479 ± 0.101

Tafel polarization measurements were used to examine the exchange current density (*J*_0_) at the electrolyte–catalyst interface (shown in Fig. 7c). The tangential slope of the Tafel curve provides information about the exchange current density (linear segments extrapolate to an intercept of log J_0_), which is closely associated with the *R*_ct_ value (Eq. (5)) [19]. As we can see in Fig. 7c, the 300 °C annealed TR-MoS_x_ electrode has a large exchange current density (*J*_0_) compared with the 250 and the 350 °C annealed samples and was comparable with the TD-Pt electrode (summarized in Table 3), which means higher electrocatalytic activity and lower charge–transfer resistance at the electrolyte–electrode interface.

### Photovoltaic Performance of DSSCs

As shown in Fig. 7d, the photovoltaic performance of DSSCs is characterized using the short-circuit current density (*J*_sc_), open-circuit voltage (*V*_oc_), fill factor (FF), and photoconversion efficiency (ɳ); these parameters are summarized in Table 3. The *J*_sc_, *V*_oc_, and FF of the DSSC with a reference TD-Pt film CE were 17.056 mA cm^−2^, 0.724 V, and 0.557, respectively, yielding a photoconversion efficiency of 6.929 %. The DSSC with the TR-MoS_x_ CE annealed at 300 °C exhibits a higher conversion efficiency (6.351 %) compared with those prepared at other annealing temperatures; the corresponding *J*_sc_, *V*_oc_, and FF were 16.905 mA cm^−2^, 0.727 V, and 0.517, respectively, which agreed with the CV and EIS measurements. It is worth noting that the values of *J*_sc_ for the TR-MoS_2_ CEs annealed at 250 and 350 °C were 15.442 and 16.063 mA cm^−2^, respectively.

As we can see, Tafel-polarization measurements (Fig. 7c) are consistent with the EIS results (showed in Fig. 7b) because *J*_0_ is inversely proportional to *R*_ct_ (Eq. (5)). The lower charge–transfer resistance (*R*_ct_) at the electrolyte–electrode interface reduces the loss during charge transportation and enhances charge collection efficiency, increasing the photocurrent (*J*_sc_) and FF of DSSCs.

5 $$ {J}_0=\frac{RT}{nF{R}_{\mathrm{ct}}} $$

From Fig. 7c, the TR-MoS_2_ electrode annealed at 300 °C also possesses the largest limiting current density (*J*_lim_), which depends on the diffusion coefficient of the I^−^/I_3_^−^ redox couple in the DSSC according to Eq. (6) [19].

6 $$ D=\frac{l}{2nFC}{J}_{\lim } $$

where *R* is the gas constant, *D* is the diffusion coefficient of the triiodide, *l* is the spacer thickness, and *F* and *n* have their normal meanings. In other words, EIS and Tafel results explain the good photovoltaic performance of DSSCs based on the TR-MoS_2_ CE annealed at 300 °C.

The values of *J*_sc_ and FF can be considered indicative of the number of edge-plane active sites for redox reactions[3]. Although the crystallization of MoS_2_ was more complete at higher temperature, the *J*_sc_ and FF of TR-MoS_x_ annealed at 350 °C (*J*_sc_ = 16.063, FF = 0.299) are smaller than those of the TR-MoS_x_ annealed at 300 °C (*J*_sc_ = 16.905, FF = 0.517). Here, we suggest that the long-range-order nanostructure of the 350 °C annealed MoS_2_ reduces the active sites of the edge-planes.

In summary, the independent MoS_2_ nanosheets annealed at 300 °C provide the best electrocatalytic activity toward I_3_^−^ reduction. CV, EIS, and Tafel measurements suggest that the 300 °C annealing temperature should generate a larger active area. This results an excellent photovoltaic conversion efficiency (PCE) of 6.351 % under AM 1.5 illumination of 100 mW cm^−2^, up to 91.7 % of which is obtained using the conventional TD-Pt CE (PCE = 6.929 %). These results demonstrate that the 300 °C annealed TR-MoS_2_ CE has a great potential as a low-cost alternative to Pt in DSSCs.

## Conclusions

In this work, a two-dimensional nanostructure of MoS_2_ has been successfully synthesized by a low temperature TR method on FTO glass substrates. This material was also incorporated into a Pt-free DSSC for application. In the TGA results, it was found that the MoS_2_ sulfidization temperature was approximately 300 °C which provided the effective MoS_2_ phase transformation process. Additionally, XPS, TEM, and XRD indicate that the stoichiometry and crystallization of MoS_2_ were more complete at higher temperatures; however, these temperatures reduce the number of edge-plane active sites in the short-range-order nanostructure. The electrochemical analysis also showed that the 300 °C annealed TR-MoS_2_ CE provided an independent nanosheet nanostructure with numerous active sites that demonstrated Pt-like electrocatalytic activity for I_3_^−^ reduction. These short-range-order nanostructure of MoS_2_ nanosheets provided a great of edge-plane active sites to enhance the catalytic performance to increase the *J*_sc_ and *FF*, and an outstanding PCE can be obtained. Accordingly, the DSSC assembled with the 300 °C annealed TR-MoS_2_ structure exhibited an excellent PCE of 6.351 %; up to 91.7 % of which is obtained using the conventional TD-Pt CE (PCE = 6.929 %). This leads us to the conclusion that the low temperature TR-MoS_2_ CE is a promising candidate for application as a highly efficient and low-cost CE material in Pt-free DSSCs.
